# Simultaneous evaluation of substrate-dependent oxygen consumption rates and mitochondrial membrane potential by TMRM and safranin in cortical mitochondria

**DOI:** 10.1042/BSR20150244

**Published:** 2016-01-22

**Authors:** Subir Roy Chowdhury, Jelena Djordjevic, Benedict C. Albensi, Paul Fernyhough

**Affiliations:** * Division of Neurodegenerative Disorders, St Boniface Hospital Research Centre, Winnipeg, MB, Canada R2H 2A6; † Department of Pharmacology & Therapeutics, University of Manitoba, Winnipeg, MB, Canada R3E 0T6

**Keywords:** Alzheimer's disease, cortex, membrane potential, mitochondrial respiration, safranin O, TMRM

## Abstract

Mitochondrial membrane potential (mtMP) is critical for maintaining the physiological function of the respiratory chain to generate ATP. The present study characterized the inter-relationship between mtMP, using safranin and tetramethyl rhodamine methyl ester (TMRM), and mitochondrial respiratory activity and established a protocol for functional analysis of mitochondrial bioenergetics in a multi-sensor system. Coupled respiration was decreased by 27 and 30–35% in the presence of TMRM and safranin respectively. Maximal respiration was higher than coupled with Complex I- and II-linked substrates in the presence of both dyes. Safranin showed decreased maximal respiration at a higher concentration of carbonyl cyanide-4-(trifluoromethoxy)phenylhydrazone (FCCP) compared with TMRM. FCCP titration revealed that maximal respiration in the presence of glutamate and malate was not sustainable at higher FCCP concentrations as compared with pyruvate and malate. Oxygen consumption rate (OCR) and mtMP in response to mitochondrial substrates were higher in isolated mitochondria compared with tissue homogenates. Safranin exhibited higher sensitivity to changes in mtMP than TMRM. This multi-sensor system measured mitochondrial parameters in the brain of transgenic mice that model Alzheimer's disease (AD), because mitochondrial dysfunction is believed to be a primary event in the pathogenesis of AD. The coupled and maximal respiration of electron transport chain were decreased in the cortex of AD mice along with the mtMP compared with age-matched controls. Overall, these data demonstrate that safranin and TMRM are suitable for the simultaneous evaluation of mtMP and respiratory chain activity using isolated mitochondria and tissue homogenate. However, certain care should be taken concerning the selection of appropriate substrates and dyes for specific experimental circumstances.

## Abstract (precis)

Simultaneous evaluation of two mitochondrial bioenergetics parameters, respiration rates and mitochondrial membrane potential (mtMP) can be useful to determine the mitochondrial dysfunction under various pathological conditions including neurodegenerative diseases and diabetes.

## INTRODUCTION

Mitochondrial abnormalities are a major factor in the pathology of numerous human diseases, including cardiovascular, neurodegenerative, and cancer and this has triggered increasing interest in the analysis of mitochondrial functions. The mitochondrial membrane potential (mtMP) is the major component of the proton motive force generated across the inner mitochondrial membrane and a central process driving oxidative phosphorylation (OXPHOS). The efficiency of OXPHOS depends on the nature of respiratory substrates, which feed electrons to the respiratory chain at different levels. Three coupling sites (CI, CIII and CIV) are involved in respiration with CI-linked substrates, whereas only two coupling sites (CIII and CIV) are active in the pathway of electron transfer from succinate (CII-linked substrate) to oxygen. mtMP is often used as an indirect readout of mitochondrial metabolic state and activity [1,2]. However, the interaction between mtMP and respiration rate is not fully understood. Both parameters are not exclusively linked with each other but may be additionally maintained by a number of independent variables. Since mtMP is an intermediate of electron transfer and ATP synthesis, mtMP depends on and exerts control over these processes [3]. Taken together, determination of the interaction and interdependence of mtMP and mitochondrial respiration remains a challenging subject of research and application in mitochondrial biology. Several fluorescent probes [safranin, rhodamine 123, tetramethylrhodamine methyl ester (TMRM), tetramethylrhodamine ethyl ester (TMRE), JC-1] can be used to determine the mtMP in different types of cells and tissues [4–7]. Safranin and TMRM are hydrophobic cations that accumulate in mitochondria in response to negative potential generation in the energized state with a simultaneous change in absorption and fluorescence spectra. When an oxidation substrate is added to such a system, the fluorescence intensity drops in response to energization, which is due to accumulation and quenching of fluorescence of the dye in the mitochondrial matrix. The two most common fluorescent dyes, safranin and TMRM, have been used to assess the mtMP, and only one study [8] simultaneously evaluated mtMP and oxygen consumption rates (OCR) by using safranin. Krumschnabel et al. [8] described safranin's suitability for the simultaneous measurement of OCR and mtMP and also suggested the detection of mtMP needs to be validated with the quality control by respirometry for the proper interpretation of pathological conditions in terms of their impact on mitochondrial functional states. No detailed study has investigated whether TMRM can be used for the simultaneous evaluation of mtMP and OCR in isolated mitochondria.

The aim of the present study was to characterize the simultaneous inter-relationship between mtMP and mitochondrial respiratory chain activity and establish a protocol to analyse the functional aspects of mitochondrial energetics in a multi-sensor system, with mitochondrial complex specific substrates and an uncoupler in brain cortical mitochondria. As an example, we used this multi-sensor system to measure simultaneously mtMP and OCR in the cortex of triple transgenic mice (3xTg-AD), a model for Alzheimer's disease (AD). AD, the most common late onset neurodegenerative dementing disorder, is characterized by progressive neuronal loss, especially in the hippocampus and cortex [9]. Although plaques and tangles are hallmarks of the disease, emerging evidence strongly support the idea that mitochondrial dysfunction is an early event in the onset and progression of AD [10,11].

## MATERIALS AND METHODS

### Preparation of tissue homogenates and isolated mitochondria

Male Sprague–Dawley adult rats of 300–400 g were used to prepare brain cortical tissue homogenates. Triple-transgenic mouse model (3xTg-AD) were used in the present study, and possess the M146V mutation knocked into the PS1 gene and overexpress human APPswe and tauP301L [12]. This strain exhibits age-dependent neuroanatomical and cognitive parallels to AD, including plaque formation, neurofibrillary tangles and memory deficits, making it similar to AD in humans [13]. C57BL/6 mice were used as background control since our 3xTg-AD mice were maintained on C57BL/6 for eight generations. After cervical dislocation, the whole brain was dissected from the skull. Cortical tissues were rinsed in ice-cold solution containing mitochondrial isolation buffer (MIB: 70 mM sucrose, 210 mM mannitol, 5 mM HEPES, 1 mM EGTA and 0.5% (w/v) fatty acid free BSA, pH 7.2). Then tissues were homogenized with a 2 ml glass homogenizer using 10 full strokes (up and down cycles) with both large and small pestles. Mitochondria were isolated using a differential centrifugation method [14]. The homogenate was centrifuged at 800 × ***g*** for 10 min at 4°C in an Eppendorf Centrifuge 5810R using FA45-30-11 rotor (Eppendorf Canada). The pellet was removed and the supernatant was spun again at the same speed and time. The supernatant enriched with mitochondria was then spun at 8000 × ***g*** for 15 min and pellets were washed once more for 15 min in the same buffer. The pellet, representing the mitochondrial-enriched fraction, was resuspended in MIB buffer. The protein content was measured according to the method of Bradford [15]. Animal procedures followed guidelines laid down by the University of Manitoba Animal Care Committee using the Canadian Council of Animal Care guidelines.

### Measurement of mitochondrial oxygen consumption

Oxygen consumption was determined at 37°C using the OROBOROS Oxygraph-2K-Fluorescence LED2 module (OROBOROS instruments GmbH). Tissue homogenates or isolated mitochondria from cortical tissues were resuspended in KCl-enriched buffer (80 mmol/l KCl, 10 mmol/l Tris/HCl, 3 mmol/l magnesium chloride, 1 mmol/l EDTA, 5 mmol/l potassium phosphate, pH 7.4). Various substrates and inhibitors for mitochondrial respiratory chain complexes were used as described later. In the experiments where all substrates (glutamate, malate, pyruvate and succinate) were applied we used 200 μg of mitochondrial protein due to the rapid decrease in oxygen level, whereas in other experiments 300 μg of mitochondrial protein was used.

### Analysis of the mitochondrial membrane potential

mtMP was assessed with fluorescent dyes, safranin and TMRM. The O2k-Fluorescence LED2 module is equipped with filter sets for safranin (excitation at 495 nm and emission at 587 nm) and TMRM (excitation, 530±21 nm; emission, 592±22 nm) respectively. Safranin is a lipophilic cationic dye that accumulates in mitochondria according to the inside negative potential in energized mitochondria with concomitant change in absorption and fluorescence. In the course of this process, safranin undergoes a significant change in absorption and self-quenching of its fluorescence [7,16]. These spectral changes are linearly related to mtMP within certain concentrations of dye and amount of mitochondria [17,18]. Safranin dissolved in distilled water was titrated up to a final concentration of 2.5 μM. A linear increase in the fluorescence signal was detected, reflecting the concentration of safranin in the chamber. Isolated mitochondria were then added to chambers. In the absence of respiratory inhibitors, the mtMP builds up on the basis of endogenous substrates; a corresponding amount of the dye accumulates in the mitochondrial matrix, i.e. decline of fluorescence signal after initial maximal signals. A fraction of safranin binds non-specifically to the mitochondria, and therefore, the free safranin concentration is lower than the total safranin concentration added to the chamber. TMRM is a cell-permeant cationic lipophilic red fluorescent dye that is accumulated by mitochondria in proportion to mtMP. Upon accumulation of the dye it exhibits a red shift in its absorption and fluorescence emission spectrum. The fluorescence intensity is quenched when the dye is accumulated within the matrix of mitochondria. The quenching mode concentration of TMRM in isolated cortex mitochondria was reached at 2 μM of TMRM [19]. Therefore, isolated mitochondria were added to chambers prior to the titration of TMRM to a final concentration of 2 μM.

### Measurement of citrate synthase activity

To determine the level of mitochondrial mass in both samples of cortical mitochondria from age-matched controls and 3xTg mice, citrate synthase was assessed as an exclusive marker of the mitochondrial matrix. The enzymatic activity of citrate synthase was performed spectrophotometrically using a temperature controlled Ultrospec 2100 UV–visible spectrophotometer equipped with Biochrom Swift II software (Biopharmacia Biotech). The enzymatic activity of citrate synthase was determined at 25°C in a medium containing 150 mmol/l Tris/HCl (pH 8.2), 0.01% dodecyl maltoside, 0.1 mmol/l dithionitrobenzoic acid and 5 μg protein of isolated cortical mitochondria [20]. The reaction was initiated by the addition of 100 μmol/l acetyl CoA and changes in absorbance at 412 nm were measured for 1 min. This value was subtracted from the rate obtained after the subsequent addition of 0.05 mmol/l oxaloacetic acid. The enzymatic activity of cortical mitochondria from controls and 3xTg did not show significant difference (Control: 740.4±17.3 compared with 3xTg: 713.2±62.2, means ± S.E.M., *n*=3). This demonstrates the assay utilized equal amounts of mitochondria from the brain of controls and 3xTg mice.

### Chemicals

TMRM was obtained from Molecular Probes. Safranin, pyruvate, glutamate, ADP, succinate, oligomycin, carbonyl cyanide-4-(trifluoromethoxy)phenylhydrazone (FCCP), rotenone, antimycin A and all other common chemicals were purchased from Sigma–Aldrich unless stated otherwise.

### Data analysis

OROBOROS DatLab software was used to calculate the OCRs and for the graphic presentation of experimental data. Polarographic oxygen sensors monitored changes of oxygen concentration over time. The negative time derivative is accordingly corrected for instrumental background and displayed in real time as oxygen flux, simultaneously with the fluorescence signals for both chambers. This software also analysed mtMP. Both dyes accumulate in mitochondria according to the inside negative potential in energized mitochondria, with concomitant change in absorption and fluorescence, meaning that with higher potential the accumulation of the dyes in the mitochondria is higher but the fluorescence signal is lower. For calibration of the safranin or TMRM signals, the appropriate plot of the raw signal is selected and marks are set to data regions before and after stepwise addition of safranin or TMRM. The increase in fluorescence signal (less dye is accumulated in the mitochondrial matrix and thus less quenching) indicates the decrease in mtMP in mitochondria and vice versa. Datlab then calculates the parameters of a linear regression analysis through these data and the values for sensitivity as well as the coefficient of determination, *R*^2^. The signal was then normalized (0–1) to detect changes of substrates, inhibitors and uncoupler within concentrations of a minimum 0 (set as 0) to maximum 2.0 and 2.5 μM for TMRM and safranin respectively (set as 1). All experiments have been repeated at least two to three times, to confirm the observed results.

## RESULTS AND DISCUSSION

### Simultaneous evaluation of OCR and mtMP in the presence of substrates for Complex I and II using two fluorescent dyes, safranin and TMRM

**Figure 1:**
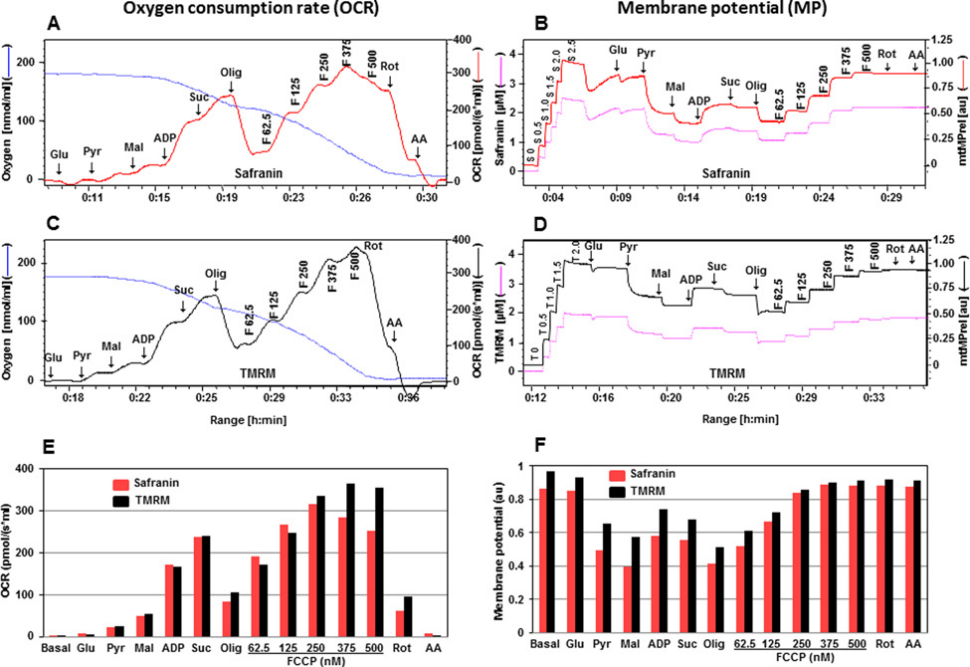
Comparison of two fluorescent dyes, safranin and TMRM, during simultaneous measurement of OCR and mtMP in isolated rat cortical mitochondria (200 μg of protein) Simultaneous assessment of OCR is depicted in **A**, **C**, **E** and mtMP in **B**, **D**, **F** with substrates glutamate (Glu: 10 mM), pyruvate (Pyr: 10 mM), malate (Mal: 2 mM), ADP (2 mM), succinate (Succ: 10 mM), oligomycin (Olig, 1 μM), FCCP (F: 62.5–500.0 nM), rotenone (Rot: 1 μM) and antimycin A (AA: 1 μM). Oxygen concentration levels (nmol/ml) are illustrated in **A** and **C** (blue thin lines; left axes) and OCR in [pmol/(s·ml)] in right axes with safranin (thick red line) and TMRM (thick black line) respectively. Fluorescent signal levels of mtMP with safranin and TMRM [(μM), purple thin lines; left axes] and relative signals, mtMP (au) in right axes with safranin (thick red line) and TMRM (thick black line) are in **B** and **D** respectively. Levels of OCR and mtMP are also represented in bar charts in **E** and **F** with safranin (red) and TMRM (black) respectively.

Mitochondrial respiration depends on a continuous supply of substrates delivering electrons and protons for maintenance of OXPHOS. Glutamate, pyruvate and malate generate NADH and this reducing agent donates electrons to Complex I and electron transport follows. Full operation of the citric acid cycle in isolated mitochondria or permeabilized tissues and cells requires addition of succinate to the conventional substrates of Complex I. Succinate supports electron flux through Complex II via flavin adenine dinucleotide (FADH_2_). Various results point to the additive effect of substrate combinations on OXPHOS capacity [8]. In the first set of experiments we used all Complex I (glutamate: 10 mM; pyruvate: 10 mM; malate, 2 mM) and Complex II-linked (succinate: 10 mM) substrates to measure OCR (Figures 1A and 1C) and determine mtMP (Figures 1B and 1D) using safranin (2.5 μM) or TMRM (2 μM). ADP (2 mM)-stimulated coupled respiration with substrates of Complex I and II was the same when measured with either safranin or TMRM. However, uncoupled respiration had a different profile. Namely, when we used safranin maximal respiratory activity was reached with lower concentrations of FCCP (375 nM) compared with TMRM (FCCP of 500 nM), after which it started declining, implying a mild toxic effect of safranin (as discussed later).

In Figures 1B and 1D, the fluorescence signal in the presence of safranin or TMRM showed a slight decrease after the addition of glutamate, indicative of increased polarization of the mitochondrial inner membrane (elevated mtMP). A sharp decrease in fluorescence signal was detected in the presence of pyruvate, reflecting an increase in mtMP and the signal was further decreased by the addition of malate. A decrease in fluorescence signal was more pronounced when safranin was used. At this point, ADP was added to activate OXPHOS and this was associated with a decrease in mtMP (increase in TMRM or safranin fluorescence). This was due to protons being shuttled through the ATP synthase and triggering phosphorylation of ADP to ATP. Consequently, there was an increase in OCR via the diminishment of the constraining effect of the proton gradient on electron transport. OCR was then enhanced by the addition of succinate with a concomitant small increase in mtMP (decrease in TMRM or safranin fluorescence). Oligomycin (1μM) induced a major rise in mtMP and a proton leak state by specific inhibition of ATP synthase activity. This blockade of ATP synthase activity fed back to significantly inhibit OCR through the build-up of the mtMP. The protonophore, FCCP was then titrated in steps of 62.5, 125, 250, 375 to 500 nM to induce a progressive depletion of mtMP by uncoupling. Finally rotenone (1 μM) and antimycin A (1 μM) were added to inhibit Complex I and III respectively.

In previous studies when safranin was introduced as an indicator of mtMP, concerns were raised regarding the possible side effects of this dye. A negative impact of safranin was noted at concentrations above 40 μM on ADP- and FCCP-stimulated respiration and at 10 μM on mitochondrial calcium cycling [21–23]. When safranin was added during Complex I-linked OXPHOS state, a strong time dependence of the inhibitory effect was observed and the dye also exerted a dose-dependent inhibitory effect, resulting in approximately 70% inhibition of Complex I-linked respiration at 4 μM [8]. Although our results indicated that the presence of safranin had a mild toxic effect on the FCCP-stimulated uncoupled respiration, the pattern of mtMP responses to mitochondrial substrates, inhibitors and the uncoupler were very similar to TMRM (Figures 1B and 1D). In comparison with TMRM, the changes in mtMP induced by substrates in the presence of safranin were greater and indicate a higher sensitivity of this dye to detect changes in mtMP (Figure 1F). These results indicate that although both fluorescent dyes are suitable for the simultaneous evaluation of OCR and mtMP with the mitochondrial substrates for Complex I and II, TMRM is a better choice for OCR measurements due to a toxic effect of safranin, but safranin has a higher sensitivity to changes in mtMP.

**Figure 2:**
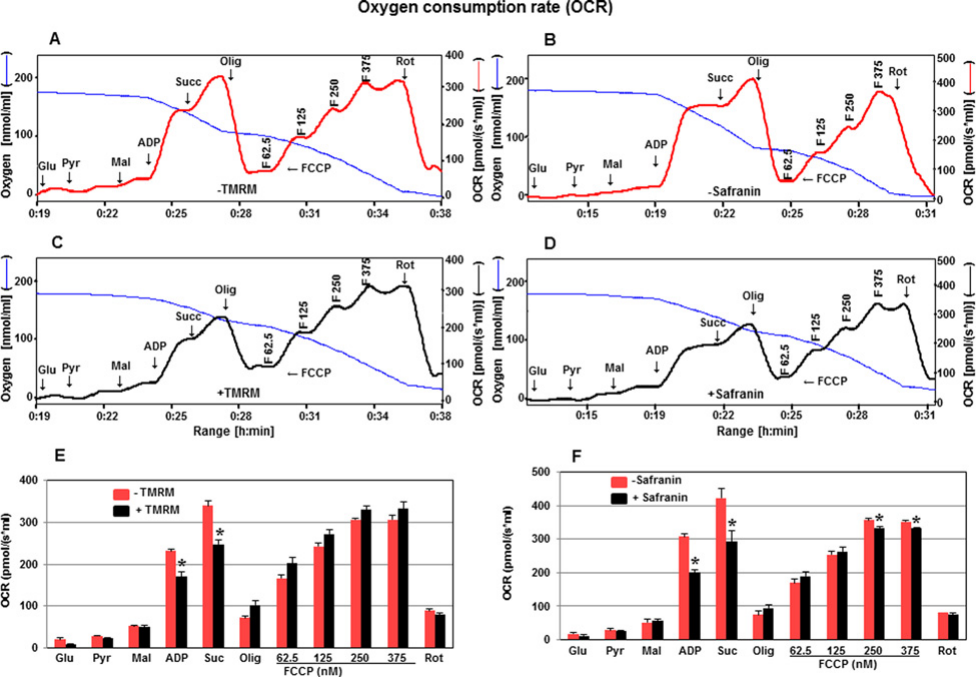
Effect of fluorescent dyes, TMRM and safranin on OCR OCR is measured in the absence or presence of TMRM (**A**, **C**, **E**) or safranin (**B**, **D**, **F**) with substrates glutamate, pyruvate, malate, ADP, succinate, oligomycin, FCCP (62.5–375 nM). Oxygen concentration levels (nmol/ml) are illustrated in **A**, **B**, **C** and **D** (blue thin lines; left axes) and OCR in [pmol/(s·ml)] in right axes in the absence (thick red line) or presence (thick black line) of TMRM (**A**, **C**) and safranin (**B**, **D**). Levels of OCR are also represented in bar charts in **E** (TMRM) and **F** (safranin) in the absence (red) and presence (black) of TMRM and safranin respectively. Values are expressed as means ± S.E.M., *n*=3. *P<0.05 compared with control (unpaired Student's *t*-test).

To assess further the toxic effect of fluorescent dyes, TMRM and safranin, on OCR in cortical mitochondria, OCR was determined in the absence or presence of these dyes (Figure 2). In the presence of TMRM (2 μM) the coupled respiration with Complex I substrates (pyruvate, glutamate, malate) or upon the addition of Complex II substrate (succinate) was decreased by 27% (Figures 2A, 2C and 2E). In the presence of safranin (2.5 μM) the coupled respiration was decreased with Complex I and Complex II substrates by 35 and 30% respectively (Figures 2B, 2D and 2F). These results suggest that both dyes most likely interfere with the function of ATP synthase since maximal respiration was not affected. Only in the case of higher concentrations of FCCP (250–375 nM), safranin decreased maximal respiration, whereas TMRM showed no toxic effect in the presence of FCCP (62.5–375.0 nM). This further confirms that TMRM is a comparatively superior choice for the simultaneous evaluation of OCR and mtMP in cortical mitochondria.

### Evaluation of OCR and mtMP in the presence of Complex I substrates, glutamate + malate compared with pyruvate + malate

In general, to assess the mitochondrial OCR related to Complex I the substrates used are glutamate + malate or pyruvate + malate. Substrate combinations of pyruvate + malate or glutamate + malate activate dehydrogenases with subsequent reduction of NADH, which then donates electrons to Complex I and then electrons passage down the thermodynamic cascade through the Q cycle of the electron transfer system to Complex IV and to the terminal acceptor, O_2_. In human skeletal muscle, conventional assays with pyruvate + malate or glutamate + malate yield submaximal oxygen consumption fluxes at 0.50–0.75-fold of the full capacity of OXPHOS [24]. In human skeletal muscle mitochondria, respiration with glutamate + malate in the presence of ADP is identical or 10% higher than with pyruvate + malate [25,26]. These results on isolated mitochondria agree with results on permeabilized muscle fibres [27,28]. In fibroblasts, glutamate and malate support a higher respiratory flux compared with pyruvate and malate [29].

**Figure 3:**
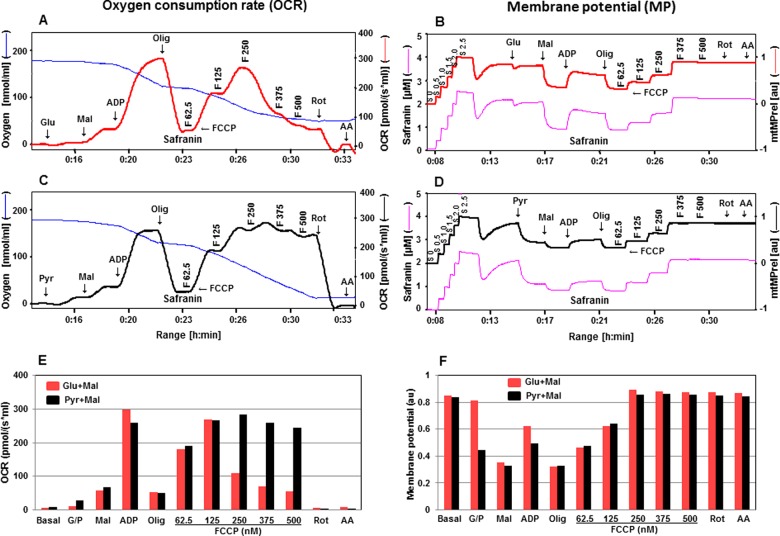
Simultaneous evaluation of OCR and mtMP in isolated rat cortical mitochondria in the presence of safranin with substrates Glu + Mal compared with Pyr + Mal (300 μg of protein) OCR in the presence of safranin is presented in **A** with substrates glutamate and malate and in **C** with pyruvate and malate with sequential addition of ADP, oligomycin, FCCP, rotenone and antimycin A (see Figure 1 for details about concentrations of these chemicals). Oxygen concentration levels (nmol/ml) are illustrated in **A** and **C** (blue thin lines; left axes) and OCR in [pmol/(s·ml)] in right axes with Glu + Mal (thick red line) and Pyr + Mal (thick black line) respectively. Fluorescent signal levels of mtMP with Glu + Mal and Pyr + Mal [(μM), purple thin lines; left axes] and relative signals, mtMP (au) in right axes with Glu + Mal (thick red line) and Pyr + Mal (thick black line) are in **B** and **D** respectively. Levels of OCR and mtMP are also represented in bar charts in **E** and **F** with Glu + Mal (red) and Pyr + Mal (black) respectively.

**Figure 4:**
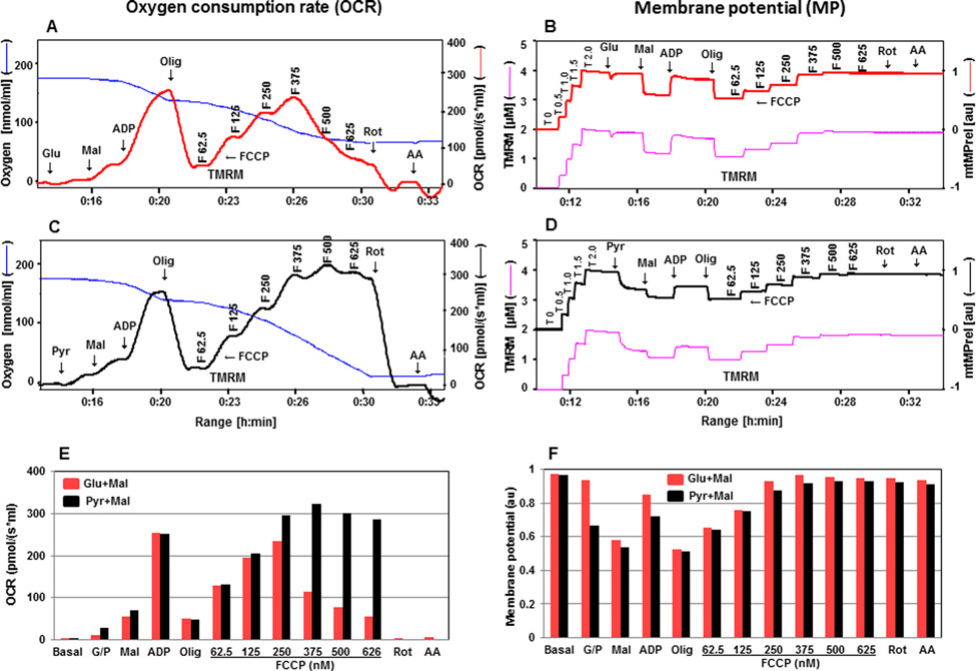
Simultaneous evaluation of OCR and mtMP in the presence of TMRM with substrates Glu + Mal compared with Pyr + Mal OCR in the presence of TMRM are mentioned in **A** with substrates glutamate and malate and in **C** with pyruvate and malate with sequential addition of ADP, oligomycin, FCCP, rotenone and antimycin A (see Figure 1 for details about concentrations of these chemicals). Oxygen concentration levels (nmol/ml) are illustrated in **A** and **C** (blue thin lines; left axes) and OCR in [pmol/(s·ml)] in right axes with Glu + Mal (thick red line) and Pyr + Mal (thick black line) respectively. Fluorescent signal levels of mtMP with Glu + Mal and Pyr + Mal [(μM), purple thin lines; left axes] and relative signals, mtMP (au) in right axes with Glu + Mal (thick red line) and Pyr + Mal (thick black line) are in **B** and **D** respectively. Levels of OCR and mtMP are also represented in bar charts in **E** and **F** with Glu + Mal (red) and Pyr + Mal (black) respectively.

The simultaneous response of OCR and mtMP to alterations in mitochondrial function was further elaborated utilizing either glutamate + malate or pyruvate + malate in the presence of safranin or TMRM (Figures 3 and 4). Our results showed that the responses of OCR and mtMP were higher after the addition of pyruvate compared with glutamate. One possible explanation could be that the transport of pyruvate is partially non-carrier-mediated if the concentration of pyruvate is above 5 mM [30]. However, these responses equalized after the addition of malate. OCR was more stable with respect to responses to uncoupling by FCCP in the presence of pyruvate and malate compared with glutamate and malate (Figures 3 and 4A, 4C, 4E). OCR driven by glutamate and malate significantly declined at FCCP concentrations of 250 nM and above (with safranin) or 375 nM and above (with TMRM); both these concentrations of safranin and TMRM maximally uncoupled the system as defined by the fluorescence signals. In contrast, OCR in the presence of pyruvate and malate was quite stable up to a 500 nM concentration of FCCP (with both fluorescent dyes). Further dissection of Complex I-linked substrates revealed that in the presence of glutamate maximal respiration was not sustainable at higher FCCP concentrations. The glutamate–aspartate carrier catalyses the electrogenic antiport of glutamate^−^ + H^+^ for aspartate^−^ and proton uptake did not occur in the presence of an uncoupling agent [31]. Gnaiger [30] also suggested that the uncoupling effect may be impaired due to the symport of glutamate^−^ + H^+^ and the fact that glutamate–aspartate antiport is not electroneutral. On the other hand, H^+^/anion symport is equivalent to OH^−^/anion antiport in the pyruvate carrier. The characteristics of glutamate–aspartate and pyruvate carriers may explain the different effects on OCR upon the FCCP titration in the presence of pyruvate + malate or glutamate + malate. Our data also showed that when pyruvate + malate were used safranin did not show the toxic effect previously described, suggesting that the inhibitory effect of safranin targets a component of the respiratory chain downstream from Complex I. mtMP levels in the presence of either dye showed similar responses to substrates, whether glutamate + malate or pyruvate + malate were used (Figures 3 and 4B, 4D, 4E).

### Simultaneous estimation of OCR and mtMP with Complex II-dependent substrates

**Figure 5:**
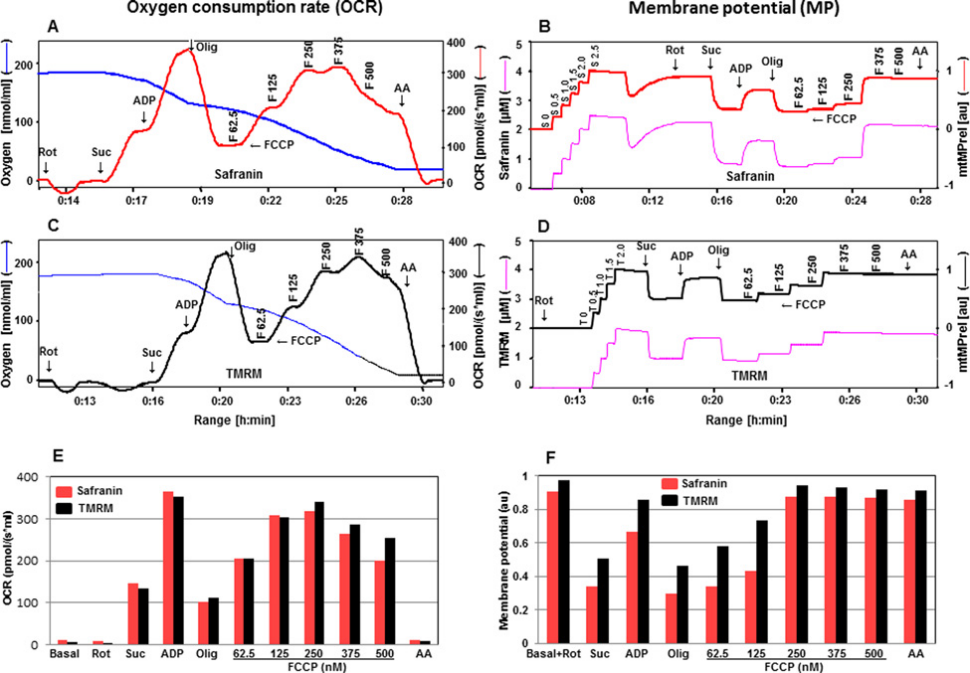
Assessment of OCR and mtMP in the presence of safranin or TMRM with succinate Simultaneous assessment of OCR is depicted in **A**, **C**, **E** and mtMP in **B**, **D**, **F** with mitochondrial substrates and inhibitors (Rot: 1 μM; Suc: 10 mM; ADP: 2 mM; Olig: 1 μM; F: 62.5–500.0 nM; AA: 1 μM). Oxygen concentration levels (nmol/ml) are illustrated in **A** and **C** (blue thin lines; left axes) and OCR in [pmol/(s·ml)] in right axes with safranin (thick red line) and TMRM (thick black line) respectively. Fluorescent signal levels of mtMP with safranin and TMRM [(μM), purple thin lines; left axes] and relative signals, mtMP (au) in right axes with safranin (thick red line) and TMRM (thick black line) are in **B** and **D** respectively. Levels of OCR and mtMP are also represented in bar charts in **E** and **F** with safranin (red) and TMRM (black) respectively.

Complex II contains succinate dehydrogenase which is the only membrane-bound enzyme that is a member of the citric acid cycle and that exists as a component of the mitochondrial electron transport chain. Succinate in the presence of rotenone, the specific inhibitor of Complex I, supports electron flux through Complex II via electron donation from FADH_2_. With succinate and ADP the levels of OCR in the presence of both fluorescent dyes were similar (Figures 5A, 5C and 5E). The pattern of mtMP in the response to substrates, inhibitors and uncouplers were also very comparable (Figures 5B, 5D and 5E). When succinate is added without rotenone, oxaloacetate is formed from malate through the action of malate dehydrogenase. Oxaloacetate cannot enter through the mitochondrial inner membrane, thus it accumulates, and is a potent competitive inhibitor of succinate dehydrogenase. Thus OCR with succinate in the presence of rotenone was 2-fold higher than without rotenone (results not shown).

### Evaluation of OCR and mtMP in isolated cortical mitochondria compared with cortex tissue homogenates

**Figure 6:**
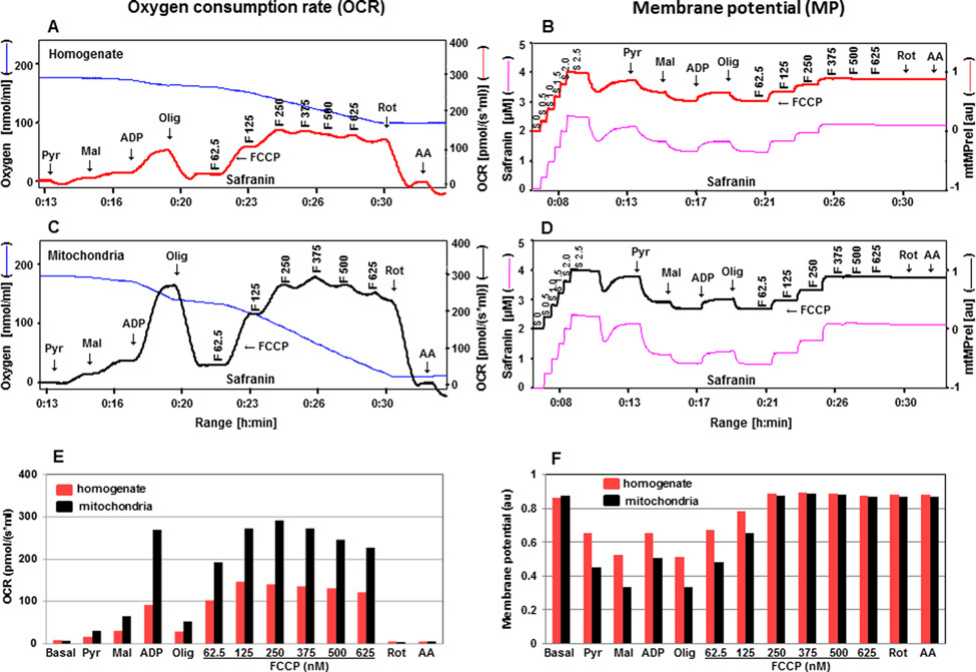
Evaluation of OCR and mtMP with substrates pyruvate and malate for homogenate compared with isolated mitochondria in the presence of safranin OCR in the presence of safranin are illustrated in **A** with cortical homogenate tissues and in **C** with cortical isolated mitochondria with sequence additions of pyruvate, malate, ADP, oligomycin, FCCP, rotenone and antimycin A (see Figure 1 for details about concentrations of these chemicals). Oxygen concentration levels (nmol/ml) are illustrated in **A** and **C** (blue thin lines; left axes) and OCR in [pmol/(s·ml)] in right axes with homogenate (thick red line) and mitochondria (thick black line) respectively. Fluorescent signal levels of mtMP with homogenate and mitochondria [(μM), purple thin lines; left axes] and relative signals, mtMP (au) in right axes with homogenate (thick red line) and mitochondria (thick black line) are in **B** and **D** respectively. Levels of OCR and mtMP are also represented in bar charts in **E** and **F** with homogenate (red) and mitochondria (black) respectively.

**Figure 7:**
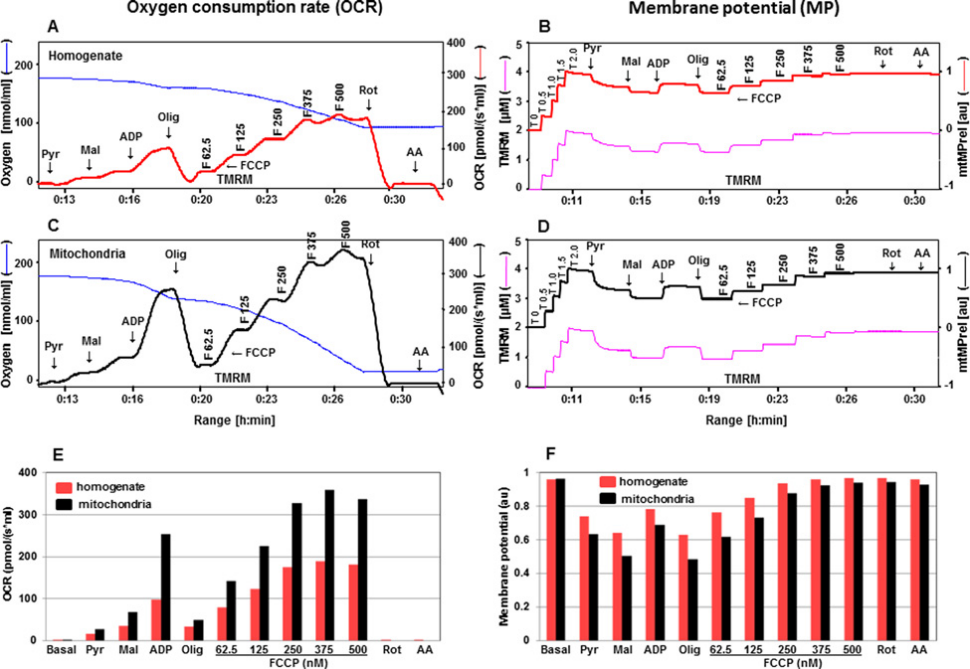
Estimation of OCR and mtMP with substrates pyruvate and malate for homogenate compared with isolated mitochondria in the presence of TMRM OCR in the presence of TMRM are depicted in **A** with cortical homogenate tissues and in **C** with cortical isolated mitochondria with sequence additions of pyruvate, malate, ADP, oligomycin, FCCP, rotenone and antimycin A (see Figure 1 for details about concentrations of these chemicals). Oxygen concentration levels (nmol/ml) are illustrated in **A** and **C** (blue thin lines; left axes) and OCR in [pmol/(s·ml)] in right axes with homogenate (thick red line) and mitochondria (thick black line) respectively. Fluorescent signal levels of mtMP with homogenate and mitochondria [(μM), purple thin lines; left axes] and relative signals, mtMP (au) in right axes with homogenate (thick red line) and mitochondria (thick black line) are in **B** and **D** respectively. Levels of OCR and mtMP are also represented in bar charts in **E** and **F** with homogenate (red) and mitochondria (black) respectively.

Tissue homogenates are isolated and used routinely for measuring mitochondrial enzyme activities, but evaluation of mitochondrial respiration and mtMP have been primarily determined in isolated mitochondria. Recently, Pecinova et al. [32] demonstrated that the analysis of homogenates allows for a highly sensitive determination of mitochondrial functions, comparable with those obtained with isolated mitochondria. The use of homogenate avoids the loss of the majority of the mitochondria during isolation, takes less time for preparation and there is no necessity for centrifugation steps. Here we have assessed the functional parameters, OCR and mtMP, using safranin and TMRM, in cortex tissue homogenates and have compared with isolated cortical mitochondria. ADP-stimulated coupled respiration with pyruvate and malate in isolated mitochondria was 2.5–3.0-fold higher compared with tissue homogenate (Figures 6 and 7A, 7C, 7E). The maximal respiratory chain activity was 2-fold higher in isolated mitochondria as compared with homogenate tissue. The extent of mtMP response to substrates was more clearly defined in isolated mitochondria compared with homogenate tissue (Figures 6 and 7B, 7D, 7F). Although using isolated mitochondria did provide some quantitative advantages compared with use of homogenates, in general, the qualitative nature of the response to substrates and uncoupling with FCCP were essentially the same. Thus, tissue homogenates are a perfectly valid source of mitochondrial material for studying mitochondrial function in the Oroboros system with combinations of these substrates and fluorescent dyes. This could provide additional opportunities when the desire is to study mitochondrial function in small tissue samples (for example, mouse tissues such as peripheral ganglia and nerve).

### Levels of OCR and mtMP are decreased in cortical mitochondria from 3xTg-AD mice

**Figure 8:**
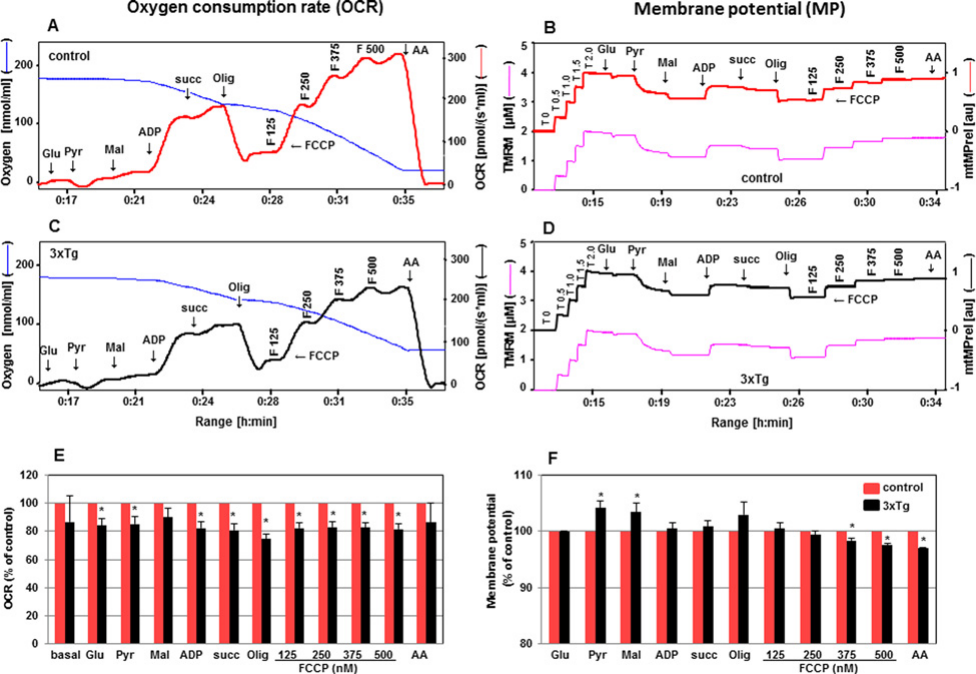
Determination of OCR and mtMP in cortex of mitochondria from 3xTg-AD mice Simultaneous evaluation of OCR and mtMP in the presence of TMRM is described in cortical mitochondria isolated from control (**A**, **B** in red) and 3xTg-AD (**C**, **D** in black) 6-month-old male mice. Changes of OCR and mtMP in 3xTg-AD mice (black) compared with control (red) are expressed in % in bar charts **E** and **F** respectively. Values are expressed as means ± S.E.M., *n*=3. *P<0.05 compared with control (unpaired Student's *t*test).

To show the suitability of simultaneous measurement of OCR and mtMP, and comparison of these two bioenergetics parameters under pathological conditions, OCR and mtMP have been assessed in cortical mitochondria from 6-month-old male 3xTg-AD mice and their age-matched controls (C57BL/6). Impaired mitochondrial function, decreased activity of respiratory chain enzymes, generation of reactive oxygen species (ROS) [33–35] and accumulation of Aβ within mitochondria [36,37], have been reported in the AD brain, as well as in the Tg-AD mouse models. Our data show significantly decreased OCR in isolated mitochondria from 3xTg-AD mice in the presence of glutamate, pyruvate, ADP, succinate, oligomycin and uncoupler, FCCP (125–500 nM) compared with their age-matched controls. These results suggest that coupled respiration, as well as the maximal respiratory capacity of the electron transport chain have been significantly diminished in the cortex of AD mice (Figure 8). Simultaneous measurements of mtMP showed small alterations in 3xTg-AD mice compared with controls in the presence of pyruvate, malate, FCCP (375–500 nM) and antimycin A, but they were statistically significant. The increase in fluorescence signals in the presence of mitochondrial substrates, pyruvate and malate, indicated a decrease in mtMP in 3xTg mice (Figure 8F). This observation aligned with the reduced electron transport chain activity. But under certain stressed conditions as in the presence of uncoupler, FCCP (375–500 nM), the changes in mtMP revealed a higher polarization status in 3xTg mice compared with control. Previous reports of the changes in mtMP after cellular stressors have been inconsistent. Several findings provide direct experimental evidence that mtMP does not always mirror changes in mitochondrial pH and respiratory status [38]. In an attempt to resolve this issue internal calibration of mtMP has not been possible in the present study due to the inability of the high resolution Oroboros oxygraph machine to be able to detect small valinomycin-induced changes in membrane potential under a range of extra-mitochondrial potassium concentrations.

## CONCLUSIONS

The simultaneous measurement of respiration and mtMP greatly enhances the informative potential of studies of OXPHOS. Our data demonstrate that fluorescent dyes, safranin and TMRM while exhibiting a low level of toxicity, are suitable for the simultaneous evaluation of mtMP and respiratory chain activity using freshly isolated mitochondria as well as tissue homogenate. The respirometric validation with different substrates, inhibitors and dose-dependent uncoupling effects are essential for any fluorescent dyes employed to assess mtMP in any respiratory state, tissue type, and pathophysiological condition. The Complex I-dependent OCR with substrates glutamate and malate is less sustainable with the uncoupling agent compared with pyruvate and malate. We used this multi-sensor system to characterize mitochondrial bioenergetics in the cortex of AD mice. The coupled respiration and the maximal respiratory capacity of the electron transport chain, along with the membrane potential were significantly altered in the cortex of AD mice compared with age-matched controls. The simultaneous analysis of substrate-dependent mitochondrial respiration and mtMP described herein are relevant for the study of mitochondrial functions in a wide variety of settings, including diabetes and neurodegenerative diseases, and permit investigation of isolated mitochondria or tissue homogenates.

## Abbreviations

AD: Alzheimer's disease
FADH_2_: flavin adenine dinucleotide
FCCP: carbonyl cyanide-4-(trifluoromethoxy)phenylhydrazone
MIB: mitochondrial isolation buffer
mtMP: mitochondrial membrane potential
mtMP (au): mitochondrial membrane potential in arbitrary units
OCR: oxygen consumption rate
OXPHOS: oxidative phosphorylation
TMRM: tetramethyl rhodamine methyl ester
